# Enhanced antibiotic release from bone cement spacers utilizing dual antibiotic loading with elevated vancomycin concentrations in two-stage revision for periprosthetic joint infection

**DOI:** 10.1007/s00264-023-05922-7

**Published:** 2023-08-11

**Authors:** Andre Lunz, Mareike Schonhoff, Georg W. Omlor, Kevin Knappe, Yannic Bangert, Burkhard Lehner, Tobias Renkawitz, Sebastian Jaeger

**Affiliations:** 1 Department of Orthopaedics, Heidelberg University Hospital, Schlierbacher Landstr. 200a, 69118 Heidelberg, Germany; 2 Laboratory of Biomechanics and Implant Research, Department of Orthopaedics, Heidelberg University Hospital, Schlierbacher Landstr. 200a, 69118 Heidelberg, Germany; 3 Center for Orthopedics and Joint Replacement, Marienhaus Hospital St. Wendel—Ottweiler, Am Hirschberg 1, 66606, St. Wendel, Germany

**Keywords:** Periprosthetic joint infection, PJI, Spacer, Two-stage revision, Dual antibiotic loaded bone cement, dALBC

## Abstract

**Purpose:**

Antibiotic loaded bone cement spacers provide high local antibiotic concentrations, preserve bone stock, and reduce soft tissue contractions. The objective of this in-vitro study was to compare antibiotic release from spacers, aiming to discover the most optimal preparation and identify modifiable factors that can further enhance antibiotic release.

**Methods:**

Six distinct spacer preparation were created using three different bone cements and manual incorporation of antibiotics. During a six-week period, the release of antibiotics from each spacer was measured individually at ten predetermined time points using a chemiluminescent immunoassay.

**Results:**

Manually adding 4 g of vancomycin to every 40 g of “Palacos R + G” yielded the most favorable release profile. Throughout all preparations, antibiotic release consistently and significantly decreased over the six-week period. When incorporating a higher concentration of vancomycin, a significantly higher cumulative release of vancomycin was observed, with varying effects on the release of gentamicin. The choice of bone cement had a significant impact on antibiotic release.

**Conclusion:**

To enhance antibiotic release from spacers, surgeons should manually incorporate high antibiotic concentrations into the most appropriate bone cement and keep the interim period as short as possible. Specifically, we suggest manual incorporation of 4 g of vancomycin to every 40 g of gentamicin premixed "Palacos R + G" to create bone cement spacers.

## Introduction

A periprosthetic joint infection (PJI) is one of the most serious complications after total joint replacement. According to the 2022 German Arthroplasty Registry Report (EPRD) it is the second most frequent reason for revision surgery [1]. The two-stage approach is still considered the “gold standard” treatment in the setting of most chronic PJIs [2, 3]. During first-stage surgery, the infected endoprosthesis is removed and thorough debridement is performed. After approximately six weeks, the two-stage procedure is completed with another round of thorough debridement and the insertion of a new endoprosthesis [4]. In the interim period the application of an antibiotic loaded bone cement spacer not only preserves the surrounding soft tissue and bone stock, but also results in high antibiotic concentrations within the infected joint, effectively supporting eradication of the infection [5–9]. Local antibiotic release has a major advantage, as it achieves high concentrations within the joint, while keeping systemic concentrations and thus the rate of adverse effects low [10–12]. Despite the clinical relevance and widespread use of antibiotic loaded bone cement spacers, there is limited data on antibiotic release from different preparations [13, 14]. Considering the existing literature, the practice of loading spacers with the vancomycin-gentamicin combination appears to be widely employed and highly effective against the majority of causative pathogens [10, 15–20]. But nevertheless, there are no specific recommendations available to guide surgeons in deciding which particular bone cement and antibiotic concentrations should be used for spacer fabrication. Therefore, this study aimed to examine and compare the antibiotic release of six different preparations of vancomycin and gentamicin loaded bone cement spacers over a six-week period. The main objective of this study was to determine the most ideal composition of a drug-eluting dual antibiotic loaded bone cement (dALBC) for spacer construction based on our findings. Our secondary goal was to identify modifiable factors that could enhance the release of antibiotics from bone cement spacers in general.

## Materials and methods

An ethics approval was not necessary for this in vitro study, which analyzed the release of antibiotics from six different preparations (Groups A-F; Table 1) made of three different bone cements: “Copal spacem” (Heraeus Medical, Wehrheim, Germany): designed for spacer fabrication without any premixed antibiotics; “Copal G + V” (Heraeus Medical, Wehrheim, Germany): specifically formulated for revision surgery with 0.5 g of gentamicin and 2 g of vancomycin per 40 g of bone cement; “Palacos R + G” (Heraeus Medical, Wehrheim, Germany): a standard bone cement used in primary total joint replacement, containing 0.5 g of gentamicin.

**Table 1:** Comparison of the six different bone cement preparations. Antibiotic dosage is given per 40 g of bone cement. (genta = gentamicin; vanco = vancomycin)

Group	Bone cement	Premixed genta	Premixed vanco	Addition of genta	Addition of vanco	Total genta	Total vanco
A	Copal spacem	-	-	0.5 g	2 g	0.5 g	2 g *(low)*
B	Copal spacem	-	-	0.5 g	4 g	0.5 g	4 g *(high)*
C	Palacos R + G	0.5 g	-	-	2 g	0.5 g	2 g *(low)*
D	Palacos R + G	0.5 g	-	-	4 g	0.5 g	4 g *(high)*
E	Copal G + V	0.5 g	2 g	-	-	0.5 g	2 g *(low)*
F	Copal G + V	0.5 g	2 g	-	2 g	0.5 g	4 g *(high)*

**Fig. 1:**
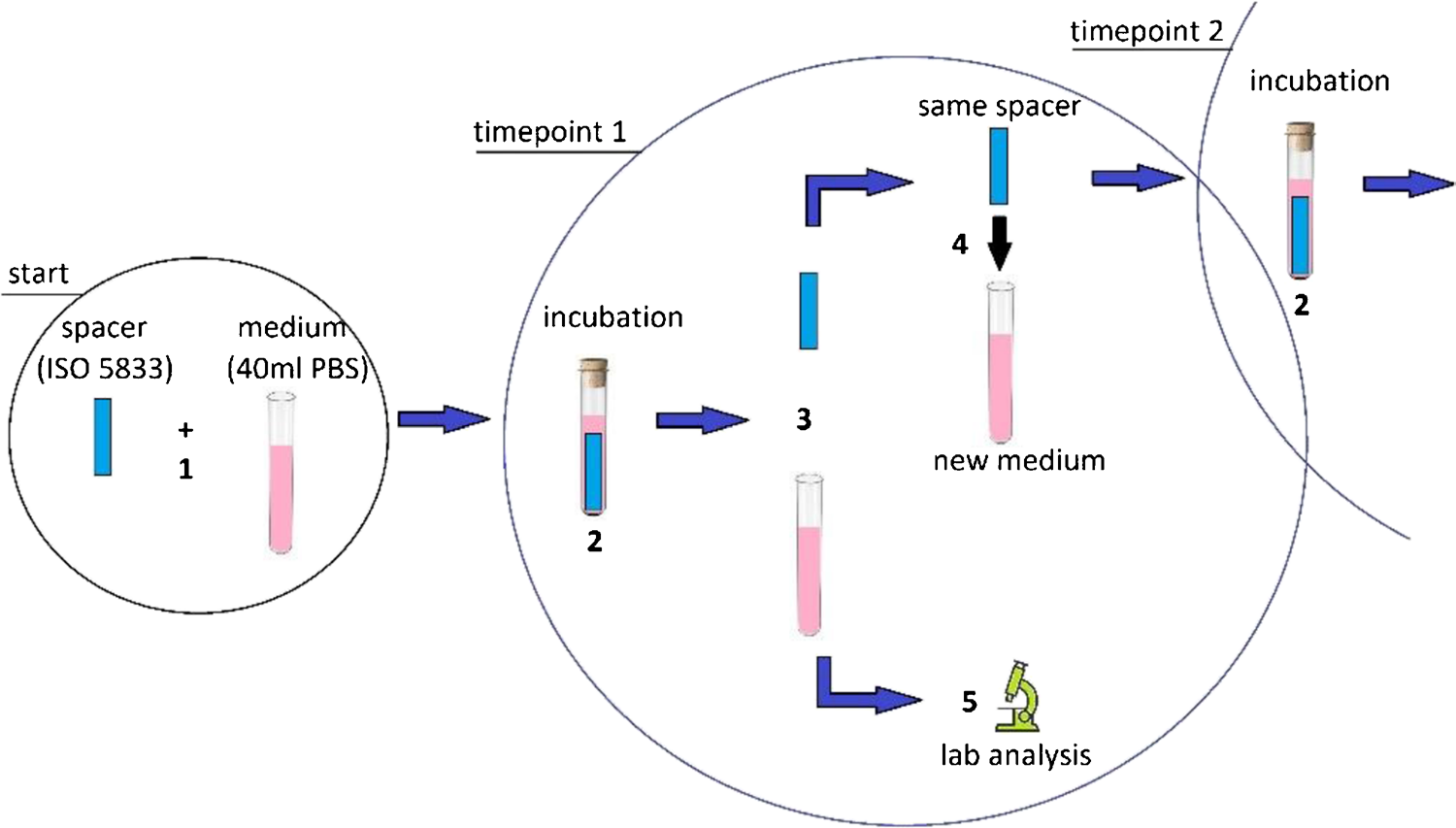
Experimental set-up. (1) Each of the 30 dALBC spacers is immersed individually in a test tube containing 40 ml of PBS. (2) Incubation is initiated. (3) At 10 predetermined time points, the specimens are taken out from the test tubes and (4) transferred individually into new test tubes with fresh 40 ml of PBS. (5) The old test tubes are sent to the lab for analysis to determine the antibiotic concentrations of gentamicin and vancomycin within the PBS medium. Meanwhile, (2) incubation continues in new test tubes. This procedure is repeated for all time points and each spacer sample

A total of 30 specimens (5 specimens from each of the 6 different preparations) were fabricated. Each spacer contained 0.5 g of gentamicin, either premixed or manually loaded (gentamicin powder, GENAXXON bioscience, Ulm, Germany) and 2 g (“low” concentration group) or 4 g (“high” concentration group) of vancomycin hydrochloride, either premixed or manually loaded (vancomycin powder, Hikma Pharmaceuticals, London, UK) per 40 g of bone cement. Manual antibiotic loading was performed following the recommendations by Kuhn et al. [21]. The powder of the added antibiotics was thoroughly ground in a mortar and then successively added to the powder of the bone cement while stirring. All cement-mixing procedures were performed without vacuum at a room temperature of 23 ± 1 °C and humidity of at least 40%. Exactly 60 s after bone cement mixing was started, the dALBCs were applied into specifically designed molds using a cement gun. The molds were clamped for an hour to achieve complete curing of the bone cement [22]. Finally, according to DIN ISO 5833 (International Organization for Standardization, Geneva, Switzerland), the surface of all specimens was carefully smoothened and measured to fulfill the geometry requirements of a rectangular block with a length of 75 ± 0.2 mm, a width of 10 ± 0.2 mm, and a total thickness of 3.3 ± 0.2 mm [23]. All specimens were then individually immersed into 40 ml of phosphate-buffered saline (PBS) with pH 7.4 at 37 °C for incubation. The antibiotic release of all specimens was separately analyzed using the chemiluminescent immunoassay (Advia Centaur XPT, Siemens Healthineers, Germany) at ten predetermined time points: five h, one day, two days, four days, one week, two weeks, three weeks, four weeks, five weeks, and six weeks. The incubation medium (40 ml of PBS) was completely replaced at each timepoint (complete wash-out), resulting in a total of 300 samples (Fig. 1).

### Statistical analysis

All descriptive data is presented as the arithmetic mean, standard deviation and minimum and maximum. The Shapiro–Wilk test was performed to confirm normal distribution of the data. Then, a mixed ANOVA with post-hoc testing and Bonferroni correction was applied. The level of significance was set at *p* < 0.05 for all statistical tests. The statistical analyses were performed using the software SPSS (version 25.0; IBM Inc., Armonk, New York, NY, USA).

## Results

### Gentamicin release (Fig. 2 and Fig. 3)

**Fig. 2:**
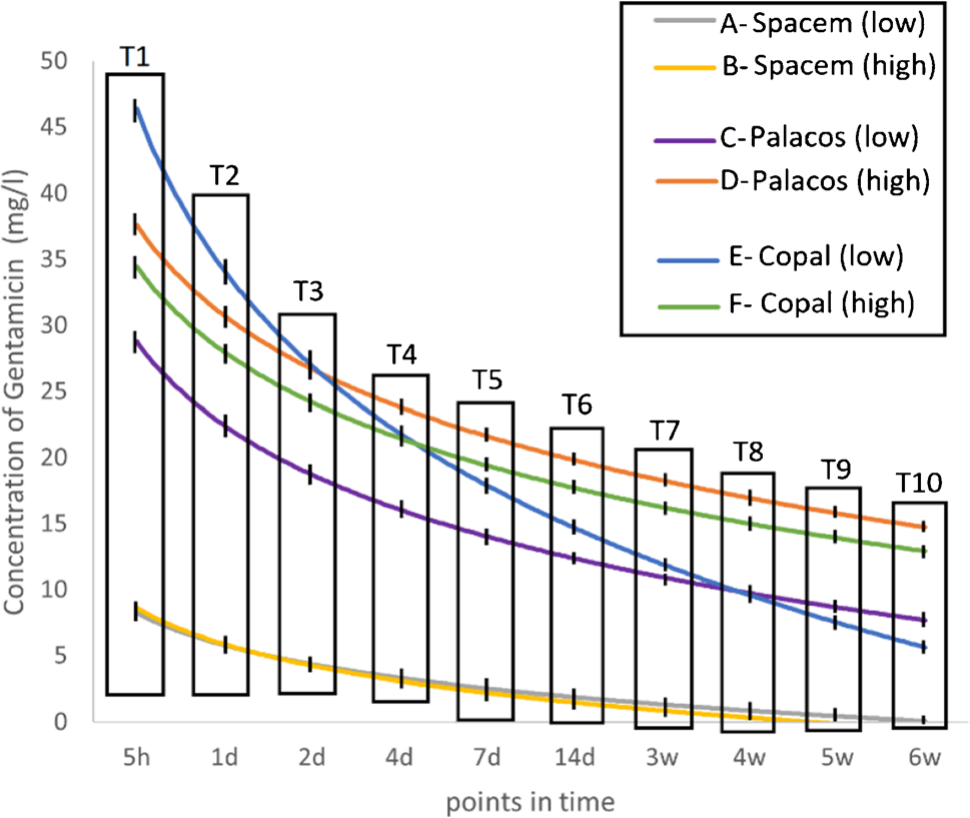
Antibiotic release of gentamicin at the predetermined timepoints T1 – T10 (shown as a logarithmic function). (low = “low” concentration; high = “high” concentration; h = hour; d = day; w = week)

**Fig. 3:**
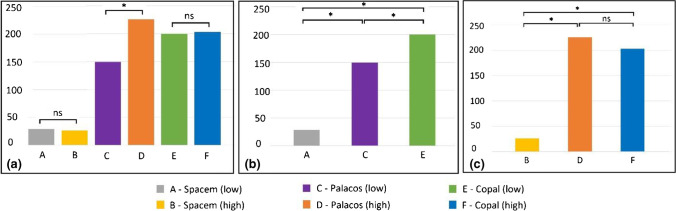
Six-week mean cumulative antibiotic release of gentamicin (mg/l). (**a**) Comparison between two formulations of the same bone cement. (**b**) Comparison between the “low” concentration preparations. (**c**) Comparison between the “high” concentration preparations. The level of significance was set at *p* < 0.05 and marked with an asterisk. (* = significance; ns = no significance; low = “low” concentration group; high = “high” concentration group)

#### Comparison between the “low” and “high” concentration preparations of the same bone cement

The higher concentration of vancomycin had a varying impact on the release of gentamicin, depending on the type of bone cement used. A statistically significant enhancement was observed for spacers composed of Palacos R + G (group C: 149.4 mg/l ± SD 15 and group D: 226.1 mg/l ± SD 13.7; *p* < 0.001, respectively), but no effect was noticed for spacers made of Copal spacem (group A: 28.9 mg/l ± SD 3.2 and group B: 26.1 mg/l ± SD 1.1; *p* = 1.0, respectively) or Copal G + V (group E: 200.2 mg/l ± SD 24.4 and group F: 203.3 mg/l ± SD 18.7; *p* = 1.0, respectively).

#### Comparison of the three “high” concentration preparations

There was no statistically significant difference in the average cumulative concentration of gentamicin over six weeks between Palacos R + G (group D) and Copal G + V (group F): 226.1 mg/l ± SD 13.7 and 203.3 mg/l ± SD 18.7; *p* = 0.38, respectively. But significant differences were found in the average cumulative concentration of gentamicin between group B (26.1 mg/l ± SD 1.1) and group D (226.1 mg/l ± SD 13.7) (*p* < 0.001), as well as between group B (26.1 mg/l ± SD 1.1) and group F (203.3 mg/l ± SD 18.7) (*p* < 0.001).

### Vancomycin release (Fig. 4 and Fig. 5)

**Fig. 4:**
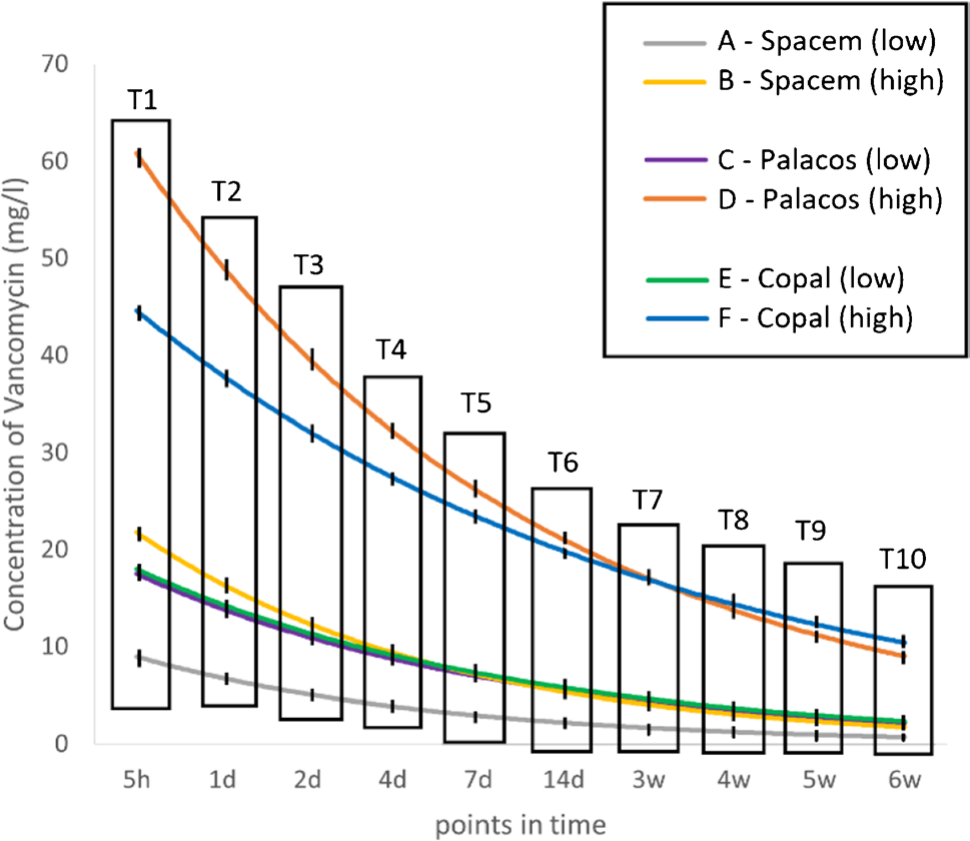
Antibiotic release of vancomycin at the predetermined specific timepoints T1 – T10 (shown as a logarithmic function). (low = “low” concentration; high = “high” concentration; h = hour; d = day; w = week)

**Fig. 5:**
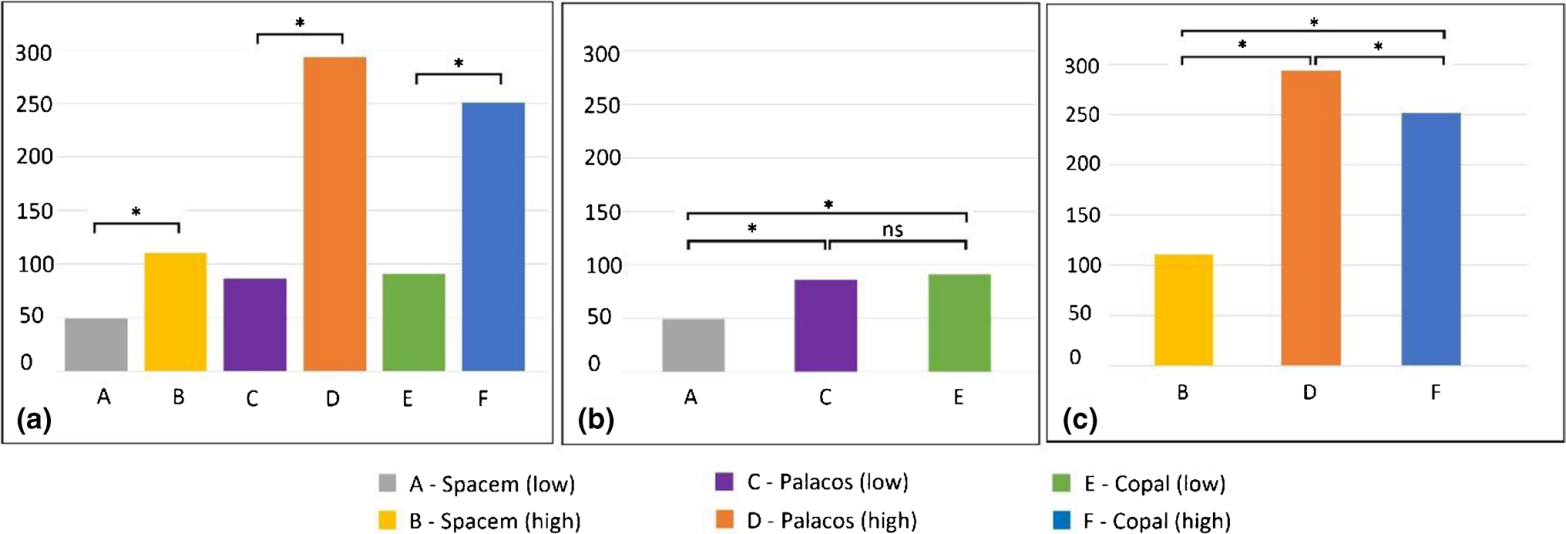
Six-week mean cumulative antibiotic release of vancomycin (mg/l). (**a**) Comparison between the two formulations of the same bone cement. (**b**) Comparison between the “low” concentration preparations. (**c**) Comparison between the “high” concentration preparations. The level of significance was set at *p* < 0.05 and marked with an asterisk. (* = significance; ns = no significance; low = “low” concentration group; high = “high” concentration group)

#### Comparison between the “low” and “high” concentration preparations of the same bone cement

The "high" concentration groups, using 4 g of vancomycin powder per 40 g of bone cement, significantly outperformed the "low" concentration groups, using 2 g of vancomycin independent of the used bone cement. There was a significant difference in the 6-week mean cumulative release of vancomycin between group A (49.3 mg/l ± SD 2.6) and group B (110.2 mg/l ± SD 5; *p* < 0.001), also between group C (86.2 mg/l ± SD 8.8) and group D (293.5 mg/l ± SD 14.5; *p* < 0.001), and group E (91 mg/l ± SD 2.5) and group F (251.2 mg/l ± SD 13.8; *p* < 0.001).

#### Comparison of the three “high” concentration preparations

The highest six-week mean cumulative release of vancomycin was observed in spacers of group D (293.5 mg/l ± SD 14.5). They significantly outperforming spacers of group F (251.2 mg/l ± SD 13.8; *p* < 0.001) and group B (110.2 mg/l ± SD 5; *p* < 0.001). There were also significant differences in the six-week mean cumulative release of vancomycin between group B (110.2 mg/l ± SD 5) and group F (251.2 mg/l ± SD 13.8) (*p* < 0.001).

## Discussion

In 1983, Insall et al. introduced the two-stage approach, which is still regarded as the most effective treatment for chronic PJIs [15, 24]. Critics of the two-stage approach argue that the six-week gap between stages leads to relevant mobility issues, patient discomfort, and pain. Additionally, the second-stage reimplantation is often complicated by the development of soft tissue contractures and arthrofibrosis. The use of an antibiotic-loaded bone cement spacer for the interim period is a significant enhancement to the original procedure. It helps to preserve the soft tissue envelope, maintains leg length, and provides a high dose of antibiotics locally [25–27]. Today, clinicians often use dALBC spacers, which effectively target an even broader range of pathogens. But an increasing number of surgeons hold the belief that commercially premixed dALBCs often lack adequate antibiotic concentrations to achieve a long-lasting bactericidal effect in the setting of PJIs. Because of this argument, the limited availability and high costs of commercially available dALBCs, it has become a common practice among many surgeons to manually add antibiotics to bone cements when constructing spacers for the interim period [5, 7, 15, 28, 29]. Despite the widespread clinical use of dALBC spacers, there are currently no specific national or international recommendations regarding the selection of particular bone cements, antibiotics, or antibiotic dosages for the construction of spacers. Consequently, the main objective of this study was to compare antibiotic release of different dALBC spacers. We have demonstrated that manual incorporation of 4 g of vancomycin to every 40 g of gentamicin premixed "Palacos R + G” resulted in the most ideal release profile over a period of six weeks. Furthermore, we have identified three key factors that surgeons can modify to enhance the release of antibiotics from dALBC spacers.

In accordance with previous studies, we have demonstrated that incorporating the same concentration of antibiotics into different bone cements results in significant variations in antibiotic release [30–32]. Additionally, we have demonstrated that antibiotic release consistently decreases even over the course of six weeks. Therefore, choosing the most appropriate bone cement and keeping the interim period short can have a great impact on local antibiotic concentrations and therefore infect eradication.

Frew et al. compared antibiotic release from manually prepared vancomycin-containing formulations with commercially available vancomycin-impregnated bone cements and suggests using spacers with manual incorporation of 2 g of vancomycin per 40 g of bone cement [29]. To the best of our knowledge, there are no commercially available premixed bone cements containing more than 2 g of vancomycin per 40 g of bone cement. But according to our results the "high" concentration groups, using 4 g of vancomycin powder per 40 g of bone cement, significantly outperformed the "low" concentration groups, using 2 g of vancomycin. This is objective evidence that commercially premixed dALBCs lack adequate antibiotic concentrations when used for spacer construction and therefore, we propose a new and higher standard concentration of 4 g of vancomycin per 40 g of bone cement for spacer construction. It is worth noting that the higher concentration of vancomycin had a varying impact on the release of gentamicin, depending on the type of bone cement used. A statistically significant enhancement was observed for spacers composed of “Palacos R + G”, but no effect was noticed for spacers made of “Copal spacem” or “Copal G + V”. Therefore, we recommend manually adding 4 g of vancomycin to the gentamicin premixed "Palacos R + G" to achieve a strong synergistic effect of both antibiotics. But it is important to note that manual antibiotic loading, especially with high antibiotic concentrations, can significantly decrease the mechanical strength of a bone cement, raising the risk for mechanical complications [33]. Therefore, from a legal point of view, it is crucial to obtain a written and signed informed consent prior to using manually dALBC spacers.

The current study has limitations. First of all, it is an in-vitro study and therefore, it is unlikely that all results and conclusions can be directly transferred into clinical practice. In-vivo, it is important to differentiate between antibiotic release from the spacer and antibiotic diffusion into the surrounding tissue. An in-vitro study can only address the release from the bone cement spacer, while diffusion into surrounding tissue is virtually impossible to imitate. But even the release is influenced by many factors. Therefore, we investigated the release in a highly standardized experimental setup and reduced all influencing factors by using predefined rectangular bone cement specimens instead of real joint spacers and periodically replaced the PBS incubation medium to analyze antibiotic release. Because this experimental set-up clearly does not resemble reality, we cannot definitively say that the release behaves exactly the same way in-vivo. However, it is reasonable to assume that reduced release under best-case conditions, as in our set-up, also demonstrates reduced releases in-vivo. Due to the in-vitro nature of this study, potential systemic complications arising from enhanced antibiotic release from spacers were not investigated. Therefore, it is necessary to address these issues in future clinical studies. Furthermore, our study design does not account for the potential influence of wear caused by articulating spacers on the release of antibiotics. Therefore, it is crucial to investigate this aspect in forthcoming research endeavors. Finally, our study focused on three bone cements loaded with gentamicin and vancomycin in two different concentrations. Future research should explore other cements, antibiotics, and concentrations. Nevertheless, the tested preparations are commonly used in clinical practice. Although all identified differences are statistically significant, their exact clinical impact has yet to be determined. Still, we believe, it is imperative for orthopedic surgeons to possess a comprehensive understanding of important modifiable factors and their demonstrated impact on antibiotic release from bone cement spacers.

## Conclusion

In the management of chronic PJIs, it is common practice to manually incorporate antibiotics into bone cements during the construction of spacers in the operating theater. In this in-vitro study, we have identified three key factors that surgeons can modify to influence the release of antibiotics from spacers: Firstly, surgeons should keep the duration of the interim period as short as possible to effectively support infect eradication through high local antibiotic release. Secondly, the decision for a particular bone cement has significant impact on antibiotic release. And thirdly, dual antibiotic loading exhibits a synergistic effect on the release of antibiotics, particularly when employed at a high antibiotic concentration in conjunction with an appropriate bone cement. Based on the results of this study, we specifically suggest manual addition of 4 g of vancomycin to every 40 g of gentamicin premixed "Palacos R + G" for the construction of spacers.

## Data Availability

Available on request.
